# Toxic metals in the regulation of epithelial–mesenchymal plasticity: demons or angels?

**DOI:** 10.1186/s12935-022-02638-3

**Published:** 2022-07-27

**Authors:** Xu-Li Chen, Yan-Ming Xu, Andy T. Y. Lau

**Affiliations:** Laboratory of Cancer Biology and Epigenetics, Department of Cell Biology and Genetics, Shantou University Medical College, Shantou, Guangdong 515041 People’s Republic of China

**Keywords:** Toxicologically relevant metals, EMT, Arsenic, Cadmium, Cobalt, Chromium, Nickel, Copper

## Abstract

Epithelial cells can trans-differentiate into motile mesenchymal cells through a dynamic process known as epithelial–mesenchymal transition (EMT). EMT is crucial in embryonic development and wound healing but also contributes to human diseases such as organ fibrosis and cancer progression. Heavy metals are environmental pollutants that can affect human health in various ways, including causing cancers. The cytotoxicity and carcinogenicity of heavy metals are complex, and studies have demonstrated that some of these metals can affect the progress of EMT. Here, we focus on reviewing the roles of six environmentally common toxic metals concerning EMT: arsenic (AS), cadmium (Cd), cobalt (Co), chromium (Cr), nickel (Ni), and copper (Cu). Noteworthily, the effects of these elements on EMT may vary according to the form, dose, and exposure time; the dual role of heavy metals (e.g., AS, Cd, and Cu) on EMT is also observed, in which, sometimes they can promote while sometimes inhibit the EMT process. Given the vast number of toxicologically relevant metals that exist in nature, we believe a comprehensive understanding of their effects on EMT is required to dictate in what circumstances these metals act more likely as demons or angels.

## Background

It is becoming very common for humans to expose to toxicologically relevant metals due to the diverse applications of metals in agriculture, medicine, household, technology, and industry [1]. Exposure to toxic metals has now permeated into all aspects of our lives, and not just from toxic waste sites or sporadic poisoning events. Thus, it is more important than ever before to pay attention to the potential adverse effects of metals on the environment as well as human health. In fact, the harmful effects of certain metals have only been noticed over the past few decades following the increase of human exposure worldwide owing to industrialization [2]. One of the reasons for slow recognition of metals’ perniciousness to human health is that the toxic effects are usually not instant and can take ages to accumulate. Therefore, even after knowing the potentially toxic effects, people are still willing to take the risks of using certain toxicologically relevant metals and alloys for the need of manufacture and ease of life. For example, dental “silver” amalgam fillings that contain about 50% elemental mercury are traditionally and are still commonly used in oral treatments despite having debatable safety issues [3].

Over the years, a great number of metallic elements have successively been proven to be carcinogenic based on epidemiological, clinical, in vitro, and in vivo studies [3]. All these metals have been classified as human carcinogens (either known or probable) by reputable organizations such as the International Agency for Research on Cancer (IARC) and the United States Environmental Protection Agency (USEPA) [1]. Epithelial–mesenchymal transition (EMT), a dynamic process where epithelial cells acquire mesenchymal features, is involved in developmental and morphogenetic processes but also contributes to human diseases such as organ fibrosis and cancer progression, especially metastasis [4, 5]. Recently, heavy metals such as arsenic (AS), cadmium (Cd), cobalt (Co), chromium (Cr), nickel (Ni), and copper (Cu) have been shown to play a role in EMT, and this review aims to provide a more holistic view of the effects of these metals in the progress of EMT.

### What is EMT? Complex regulatory networks of EMT

Epithelial cells can trans-differentiate into motile mesenchymal cells through EMT, a morphogenetic process associated with wound healing, embryonic development, tissue formation, stem cell behavior, and cancer metastasis [6]. Conversely, a reversed process of EMT, known as mesenchymal–epithelial transition (MET), occurs when mesenchymal cells loss their migratory freedom and shift toward the epithelial state [6]. The concept of EMT was first described by Professor Elizabeth D. Hay in the early 1980s, as she observed the phenotypic changes of epithelial to mesenchymal state in the primitive streak of chick embryos [7]. Since then, EMT has attracted considerable attention in the field of cell biology as well as cancer research—it is now more than 20 years since EMT was first shown to be strongly associated with cancer progression [8, 9].

**Fig. 1:**
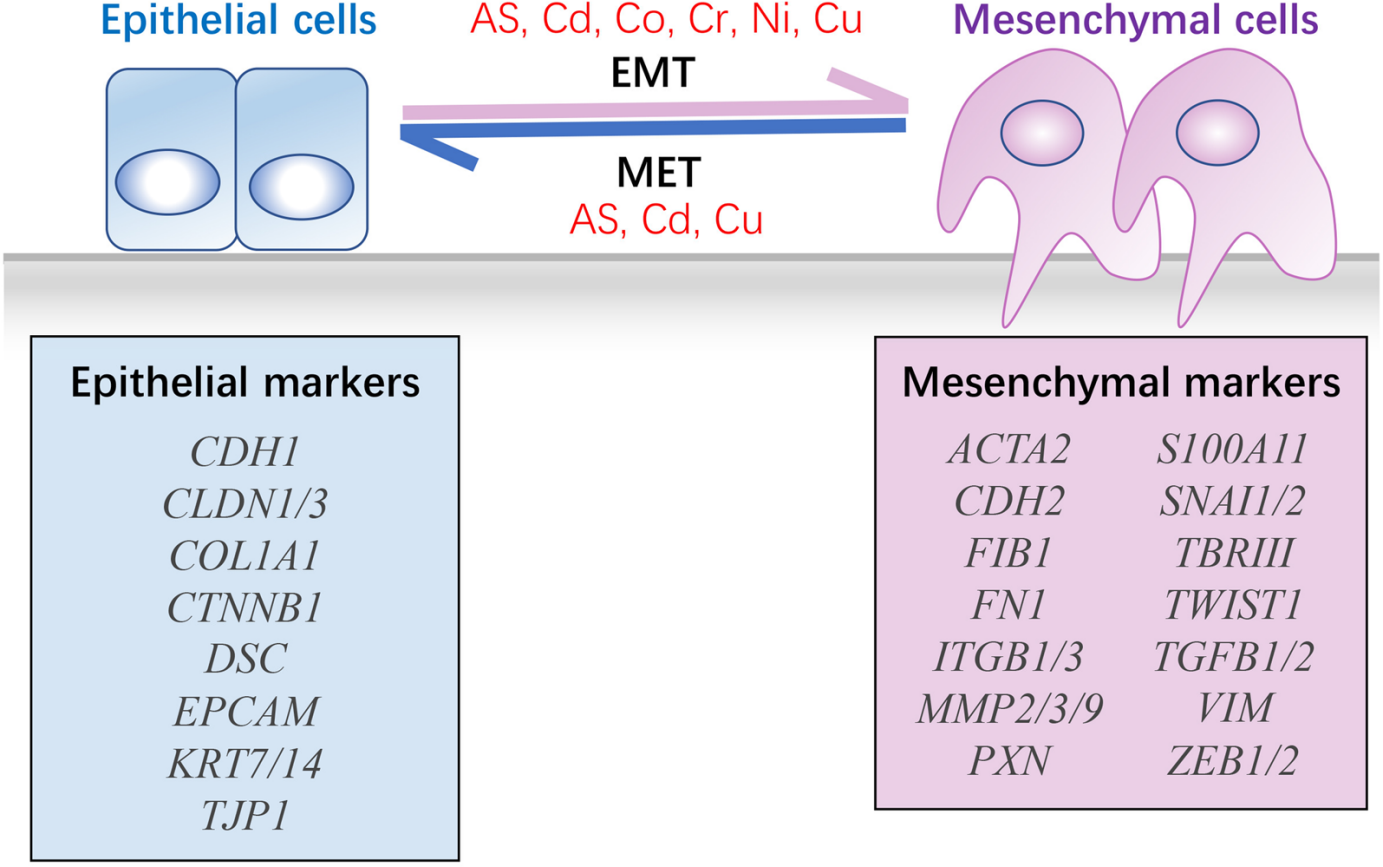
An overview of the EMT process and classic gene markers. The EMT is a dynamic and reversible process modulated by epithelial and mesenchymal marker expression: Some of the typical epithelial markers include β-catenin (*CTNNB1*) and E-cadherin (*CDH1*), whereas mesenchymal markers include N-cadherin (*CDH2*), SNAI1/2 (*SNAI1/2*), and vimentin (*VIM*). Studies have shown that toxicologically relevant metals such as AS, Cd, Co, Cr, Ni, and Cu can promote the progress of EMT, and three of these metals (AS, Cd, and Cu) may inhibit EMT

EMT is a complicated process involving more than hundreds of protein-coding and non-coding genes [10]. Some of these genes are selected and widely used as EMT markers, and these “classical” epithelial and mesenchymal markers are summarized in Fig. 1. In general, the simplest description of EMT from a molecular aspect is the loss of E-cadherin (*CDH1*; a cell–cell adhesion protein) and the gain of vimentin (*VIM*; a type-III intermediate filament protein) [6]. However, since EMT/MET occurs in a gradual manner, several intermediate states between the transition have been recently suggested, and these states can be classified as the partial-, incomplete-, and hybrid-EMT states in addition to the fully epithelial or mesenchymal state (reviewed in [6]). In many tumors, diverse EMT states of cancer cells have been observed, and these cells are associated with different metastatic potentials [11, 12].

The activation of the EMT transcription program is induced by signaling pathways mediated by transforming growth factor β (TGF-β), bone morphogenetic protein (BMP), Wnt/β-catenin, Notch, Hedgehog, and receptor tyrosine kinases [13, 14]. These pathways are usually triggered by various stimuli in the local microenvironment, such as growth factors, cytokines, hypoxia, and contact with the surrounding extracellular matrix [14]. Among the EMT-inducing pathways, the TGF-β signaling pathway is the most well-characterized, and it is usually activated by TGF-β superfamily ligands, including three TGF-β isoforms (β1, β2, and β3) and six BMP isoforms (BMP2 to BMP7) [14]. The Wnt/β-catenin signaling pathway is also considered to be a key signaling pathway driving EMT, which is mainly mediated by the Frizzled and low-density lipoprotein receptor-related protein (LRP) receptors. These Wnt receptor proteins can stabilize cytoplasmic β-catenin by preventing β-catenin from being phosphorylated by GSK-3β and degraded by proteasomes, resulting in the translocation of stabilized β-catenin to the nucleus and therefore activation of EMT [15]. Furthermore, in addition to the classical Wnt/β-catenin signaling pathway, Dissanayake et al. showed a noncanonical Wnt signaling pathway (Wnt5A/PKC signaling) that could also induce EMT [16].

### The effect of heavy metals in EMT–demons or angels?

Most researchers would have agreed that almost all toxicologically relevant metals are evil because these toxic metals usually promote or exacerbate the process of EMT upon long-term and short-term exposures. However, analysis of recent experimental results shows that certain toxic metals can also inhibit EMT, depending on the dose and exposure time, and therefore these metals are portrayed as a combination of angels and demons. Take AS for example, although studies have shown that AS (III) exposure promotes intestinal tumor cell proliferation and invasion associated with EMT, exposure to low levels of AS (III) may also disrupt normal wound healing and angiogenesis processes of metastatic cancer cells [17, 18]. The current knowledge regarding the EMT promoting or inhibitory effects of AS, Cd, Co, Cr, Ni, and Cu is summarized in Table 1.

**Table 1:** An overview of the effect of the six toxicologically relevant metals in EMT

Element	Form	Promotes EMT	Inhibits EMT
Arsenic	AsO_2_^-^	Yes	ND
	ATO	Yes	Yes
Cadmium	Cd^2+^	Yes	Yes
Cobalt	Co^2+^	Yes	ND
Chromium	Cr^6+^	Yes	ND
Nickel	Ni^2+^	Yes	ND
Copper	Cu^2+^	Yes	Yes

*Yes* reported in the literature, *ND* no data (no relevant data was available at the time of this publication)

### Arsenic

AS is widely distributed in the environment due to its natural existence and industrial and medical applications. The major inorganic forms of AS are the pentavalent arsenate and trivalent arsenite. The organic forms include the methylated metabolites monomethylarsonous acid (MMA), dimethylarsinic acid, and trimethylarsine oxide. Although there is AS pollution in the air, soil, and other sources, people are mainly exposed to unsafe levels of AS through contaminated drinking water [19]. Exposure to AS can cause serious health consequences, such as cardiovascular disease, conjunctival congestion, diabetes mellitus, weakness, neurological deficits, hypertension, cancer, and other chronic diseases [20].

Evidence is clear that AS can induce EMT in normal epithelial cells of various organs, even at low concentrations. For example, studies have indicated that chronic exposure to low levels of AS (1.0–2.5 μM of NaAsO_2_) resulted in human bronchial epithelial (HBE) cells to acquire stem cell-like properties and malignant transformation, in which, these changes were shown to be associated with the deletion of miR-200 family members and upregulation of miR-21 that induced EMT [21–23]. Furthermore, several experiments have demonstrated that HBE or human epidermal keratinocyte (HaCaT) cells chronically exposed to NaAsO_2_ resulted in increased IL-6 and miR-21, decreased PTEN, and activation of STAT3 and AKT signaling pathways [24–28]. Further mechanistic studies revealed that EMT activated β-catenin in AS-transformed HBE cells, which upregulated the level of angiogenic-stimulating growth factor VEGF and promoting angiogenesis [29].

In human bronchial epithelial BEAS-2B cells, chronic exposure to sub-lethal doses of NaAsO_2_ resulted in the inhibition of miR-100 expression, activation of autophagy, and induction of EMT via the MEK/ERK1/2 signaling pathway [30, 31]. It has been shown that miR-191 is a highly conserved oncogenic miRNA [32]. In human liver epithelial L-02 cells, NaAsO_2_ exposure increased the level of HIF-2α-mediated miR-191, and thus promoting EMT and cancer stem cell-like phenotypes [33]. In another study, treatment with various concentrations (0–8 μM) of NaAsO_2_ for 12 h or with 2 μM NaAsO_2_ for selected periods (0–24 h) in L-02 cells indicated that AS could increase the number of autophagosomes by blocking autophagic flux, leading to the accumulation of SQSTM1/p62 and upregulation of mesenchymal protein SNAI1 [34]. Similarly, treatment of human lung peripheral epithelial cells (HPL-1D) and human immortalized uroepithelial cells (SV-HUC-1) with low levels of NaAsO_2_ can induce EMT [35, 36]. By exposing NaAsO_2_ to renal cortex/proximal tubule (HK-2) epithelial cells for 72 h (acute), 3 months (long-term), and 6 months (chronic), Chang and Singh found that HK-2 cells could undergo neoplastic transformation through the acquisition of EMT when chronically exposed to a relatively lower concentration of AS [37]. Furthermore, they also found that long-term AS exposure could cause HK-2 cells to acquire DNA methylation-mediated fibrotic phenotypes and treatment of DNA methylation inhibitor 5-Aza-2'-dC could reverse the EMT properties [38].

In additional to normal epithelial cells, AS-induced EMT can also be observed in other cell types, including cancer cells. For instance, in colorectal cancer cell lines Caco2 and HCT116, exposure to NaAsO_2_ could induce *SEPT9* promoter hypomethylation, which further initiated EMT [39]. In another study, long-term treatment (6 months) of immortalized human keratinocytes (NHEK/SVTERT3-5) cells with AS trioxide (ATO) could induce EMT, impair differentiation of organotypic skin models, and mimic aspects of human skin derangements (e.g., Bowen’s disease) [40]. Also, EMT and global methylation changes were observed in human cervical cancer HeLa cells when treated with 0.5 µM NaAsO_2_ for about 45 days [41].

Although the above data have shown that AS can promote EMT and enhance tumor malignancy, some contradictory results indicate that AS can suppress EMT and may be a potent anticancer agent. Several studies have indicated that low levels of AS (III) may inhibit wound healing and angiogenesis of metastatic cancer cells [17]. In gastric cancer cell lines (AGS and MGC803), As_4_S_4_ treatment could upregulate the expression of miR-4665-3p, which in turn downregulated the expression of oncoprotein GSE1 and resulted in the reverse of EMT in these cell lines [42]. In another study, EMT in AGS cells was inhibited by ATO treatment (5 or 10 μM, 48 h) via the induction of SHP-1 and attenuation of p-JAK2/p-STAT3 [43]. ATO was also shown to suppress EMT, tumor progression, and metastasis in Buffalo rat hepatoma cell line Mca-Rh7777 by inhibiting TWIST activation [44]. Furthermore, ATO could weaken the invasiveness of chondrosarcoma cells and reverse the cells to more epithelial states by increasing the expression of miR-125b, a process associated with the demethylation of DNA [45]. In hepatocellular carcinoma cell lines, ATO treatment was able to inhibit EMT by suppressing the expression of PKM2 via the induction of anticancer lncRNA MEG3 [46]. Overall, based on these results, we can observe that exposure to AS could promote angiogenesis and EMT, leading to malignant transformation of cells as well as enhancing the migration and invasion of tumor cells; on the other hand, short-term AS exposure, especially ATO, may inhibit tumor progression and metastasis and therefore have clinical implications. However, caution should be taken when applying AS for clinical used since the EMT inhibition properties of AS could also cause other non-cancer disorders, particularly cardiovascular diseases: it was indicated that MMA (III) could inhibit EMT of epicardial cells that result in AS-associated cardiovascular disorders [47]. Also, Allison et al. showed that NaAsO_2_ exposure could disrupt TGF-β2 signals and Smad activation, leading to the blockage of developmental EMT gene programming in murine coronary progenitor cells, but AS toxicity had no significant effect on smooth muscle differentiation [48].

### Cadmium

Cd is a toxic heavy metal with considerable effect on the environment and human health. As a naturally occurring element, the presence of Cd in the environment has been substantially magnified by industrialization and human activities. Humans are mainly exposed to Cd through a number of sources, including consumption of Cd-contaminated food, working in Cd-contaminated workplaces, and smoking of cigarettes [49]. Findings from repeated studies of occupational Cd exposure and lung cancer have concluded that Cd is a human carcinogen according to the IARC and the USEPA. Some studies have also determined that Cd exposure is associated with cancers of the prostate, kidney, liver, hematopoietic system, and stomach [50].

Given the fact that Cd exposure (even through the gastrointestinal system) is strongly correlated to lung cancer, many studies have used lung cell models to study Cd toxicity [51, 52]. In our previous studies, we found that BEAS-2B cells chronically exposed to CdCl_2_ exhibited EMT phenotype that ubiquitin carboxyl-terminal hydrolase isozyme L1, a newly identified EMT suppressor, was severely downregulated in the Cd-resistant BEAS-2B cell model [53, 54]. In another study by Tanwar et al., short-term Cd exposure (0, 2.5, 5, and 10 μM CdCl_2_ for 72 h) was able to decrease the level of miR-30 family genes and upregulated SNAI1 in human lung epithelial cells [55]. In addition to human lung cells, Cd could also induce EMT in tissues of other organs: it was shown that non-cancerous breast (MCF10A) and pancreas (hTERT-HPNE) epithelial cell lines underwent EMT after exposure to 2.5 µM CdCl_2_ for 40 weeks [56]. As a key regulator of EMT, SNAI1 was also found to be upregulated upon treatment with 1 μM or 3 μM CdCl_2_ for 4 weeks in both normal and cancer-derived breast epithelial cells [57]. Furthermore, Shan et al. showed that triple-negative human breast cancer cell line MDA-MB-231 treated with 1–3 μM of CdCl_2_ for 8 weeks resulted in the suppression of breast cancer prognostic marker ferroportin, increased intracellular iron concentration, promotion of cell proliferation and migration, and induction of EMT [58]. Similarly, in other cancer cell lines, Cd-associated EMT was observed. For example, in renal cancer Caki-1 cells, EMT was promoted by Cd treatment via upregulation of PGE2 through cAMP/PKA-COX2 signaling pathway [59]. In lung adenocarcinoma A549 cells, prolonged CdCl_2_ exposure induced EMT and malignant progression via the activation of Notch1, hypoxia-inducible factor-1α (HIF-1α), and IGF-1R/Akt/ERK/S6K1 signaling pathways [60]. The induction of EMT by Cd described above has also been supported by animal studies. In order to mimic long term and chronic Cd exposure, Chakraborty et al. used drinking water containing environmentally relevant Cd (100 mg/l of CdCl_2_) to feed mice for 12 weeks, and renal fibrosis was observed, along with activation of the Wnt pathway and increased expression of EMT-related genes in the kidney tissues [61].

Despite overwhelming evidence indicating that Cd promotes EMT, one study has suggested that “physiologically” relevant concentrations of CdCl_2_ (0.25 and 2.5 μM) can inhibit EMT in adult mammary stem cells via the inhibition of HIF-1α activity (important for human mammary stem cell proliferation and branching morphogenesis) and downregulation of EMT-associated genes (e.g., *VIM*, *ZEB1*, and *TGFBI*) [62].

### Cobalt

As a rare element, the chemical properties of Co are highly similar to iron and Ni. Co can form stable salts and complex compounds, mainly as Co (II) oxide and Co (III) oxide [63]. Humans are often exposed to a wide variety of Co compounds due to their widespread occurrence in daily life, including occupational, environmental, dietary, and medical.

CoCl_2_ is a hypoxia-mimetic agent commonly used to simulate the typical hypoxic environment of cancer cells. Therefore, it is also often used to study the role of hypoxia in cancer development [64, 65]. Studies have indicated that CoCl_2_ can promote EMT by stabilizing HIF-1α (a key hypoxia marker) in various cancer cell lines. For example, stabilization of HIF-1α was observed in human pancreatic carcinoma (MiaPaCa2) and esophageal squamous cell carcinoma (TE-1 and EC-1) cell lines treated with CoCl_2_, and it was shown that the hypoxic environment in these cell lines promoted EMT via the activation of Notch1-STAT3 signaling pathway, downregulation of E-cadherin, and increased expression of N-cadherin and SNAI1 [66, 67]. In human hepatocellular carcinoma HepG2 cell line, HIF-1α increased the level of COX-2 protein and induced EMT process to cope with hypoxic environment, leading to increased invasiveness and metastasis of the cancer cells [68].

Similarly, human lung cancer cell lines (A549 and PC9) treated with 100 µM CoCl_2_ for 24–48 h exhibited EMT phenotypes such as increased invasion and migration, and these cell lines also showed increased expressions of Netrin-1 and vimentin, activated PI3K/AKT pathway, and downregulation of E-cadherin [69]. In breast cancer, Chu et al. showed that expressions of vimentin and matrix metalloproteinases (MMP2 and MMP9) were significantly increased due to hypoxia in ductal carcinoma (MDA-MB-231) and mammary tumor (MCF7) cell lines treated with CoCl_2_ [70]. The expression of CA IX, a novel prognostic marker protein for breast cancer, was also upregulated in these breast cancer cell lines and closely related to tumor cell migration and invasion [70]. In another study, it was indicated that long-term treatment of CoCl_2_ could increase the number of polyploid giant cancer cells, and these cells could asymmetrically divide into more aggressive daughter cells in breast cancer [71]. Additionally, Lester et al. showed that expression of urokinase-type plasminogen activator receptor (uPAR) was induced by hypoxia in the breast cancer MDA-MB-231 cells. The overexpression of uPAR activated uPAR-dependent cell signaling and promoted EMT in the cancer cells, and this process could be reversed by silencing the expression of uPAR or by blocking the uPAR-activated cell signaling factors [72]. Furthermore, Thongchot et al. indicated that the HIF-1α expression in and cell migration of cholangiocarcinoma cell lines (M139 and M214) with CoCl_2_-stimulated hypoxia conditions could be suppressed by treating the cells with chloroquine [73].

Besides cancer cell lines, evidence is also clear that CoCl_2_ can induce EMT in various types of normal human cells [74, 75]. For instance, Kong et al. showed that human LO2 hepatocytes treated with 100 μM for 24 or 72 h underwent EMT, and this process could be inhibited by curcumin treatment via TGF-β/Smad signaling interference [74]. In another study, human lens epithelium cell line (SRA01/04) treated with 150 μM CoCl_2_ for 48 h exhibited EMT properties such as decreased expression of E-cadherin, increased expressions of HIF-1α and Notch1, activation of SNAI1, and enhanced cell migration [75].

### Chromium

Cr is an element naturally present in the earth's crust with several oxidation states, and the two more common ones are Cr (III) and Cr (VI) [76]. The oxidation state of Cr dictates the health hazard of Cr exposure: Cr (III) is non-toxic and good for nutrition and health whereas Cr (VI) is extremely toxic and has been classified as a group I carcinogen by the IARC and USEPA [3]. In general, humans are exposed to Cr (VI) through the ingestion of contaminated food/water and occupational inhalation [77, 78]—it is estimated that more than 300,000 workers are exposed to Cr (VI) and Cr-containing compounds in the workplace each year [79].

Exposure to Cr (VI) may induce health problems, including damage to the sperm and male reproductive system, anemia, and higher risk of cancers [1]. It was discovered that the Cr (VI) concentrations in the serum of prostate cancer patients were much higher than those of benign prostatic hyperplasia patients [80]. Further in vitro and in vivo studies revealed that exposure to low doses of Cr (VI) might affect prostate cancer progression by inducing EMT [80]. The EMT-inducing ability of Cr (VI) is not only observed in cancer cells but also in normal human cell lines. For instance, it was indicated that suppressed E-cadherin levels, increased vimentin levels, and EMT phenotypes (e.g., fibroblastoid morphology) were associated with acute and chronic K_2_Cr_2_O_7_ exposures in the BEAS-2B cells [81]. In another study, Li et al. found that K_2_Cr_2_O_7_ could increase the levels of mesenchymal protein and stem cell markers in renal epithelial cells [82].

### Nickel

Ni, as the 2nd most abundant element in the Earth’s inner core, is widely distributed in the environment, air, water, and soil [3]. Ni is used in a broad variety of metallurgical processes and as a catalyst in the chemical and food industry [83]. Humans are exposed to Ni mainly through Ni-contaminated water and food, which can cause a variety of health hazards. Depending on the dose and duration of exposure, Ni can cause cardiovascular diseases, lung fibrosis, and cancer of the respiratory tract [84, 85].

The relationships between Ni exposure and lung tumorigenesis have been assessed by several researchers. Wu et al. showed that treatment of NiCl_2_ could induce fibronectin and promote TGF-β-induced EMT by decreasing the level of TAB2 via upregulation of miR-4417, in both normal (BEAS-2B) and cancerous (A549) human lung cell lines [86]. Similarly, in another study by Jose et al., EMT was induced in the BEAS-2B cells chronically exposed to 100 μM NiCl_2_ for 6 weeks, and the persistent gene expression changes in the Ni-treated cells were examined [87]. They found that upregulation of ZEB1 was required for Ni-induced EMT, and the expression of ZEB1 was persistently activated by Ni-induced epigenetic alterations (e.g., decreased H3K27me3 levels) but not by hypoxia [87].

### Copper

As an essential metal, Cu plays key roles in many physiological functions, such as oxidation resistance, energy metabolism, neuronal function, and tissue integrity [88]. However, there is also evidence indicating that excessive Cu will induce angiogenesis because Cu can directly or indirectly regulate numerous angiogenesis-related factors [89]. In addition, recent studies have suggested that Cu could be carcinogenic, and Cu exposure may be associated with breast, lung, brain, colon, and prostate cancers [90].

In general, exposure to CuCl_2_ can lead to transactivation of EMT marker genes by increasing the activity of HIF-1α [91, 92]. Guo et al. showed that CuSO_4_ could induce EMT via activation of TGF-β1/Smad and MAPKs pathways in the lung of CuSO_4_-treated mice, resulting in pulmonary fibrosis [93]. On the other hand, Li et al. showed that the removal of Cu through the silencing of Ctr1 (a transmembrane protein responsible for cellular Cu uptake) could inhibit CoCl_2_-induced EMT via HIF-1α de-stabilization, along with SNAI1 and TWIST downregulation [90]. Therefore, it has been suggested that Cu chelators have the potential to be established as anticancer drugs worthy of clinical consideration.

Although most studies have indicated that exposure to high doses of Cu is a cancer risk, some studies have suggested the opposite effects of Cu in cancer. Specifically, it was revealed that disulfiram (DSF), an aldehyde dehydrogenase inhibitor with anticancer activity [94], displayed improved anti-angiogenic activity in a Cu-dependent manner [95]. Further study on this matter showed that DSF combined with Cu could suppress hepatic carcinoma metastasis and EMT by repressing NF-κB and TGF-β1 signaling pathways [96]. Overall, the above results indicate that the effects of Cu seem to be promiscuous as Cu exhibits carcinogenic and antitumor properties.

### Risk and opportunity

The numbers and levels of heavy metals in the living environment have risen dramatically over the years owing to a series of human activities, including technological advancement, urbanization, rapid industrialization, and unsafe agricultural practices [97]. As a result, exposure to heavy metals has become a serious global health problem as these elements can accumulate in the body and cause various human diseases, including cancers [98–100]. Currently, humans are mainly exposed to toxicologically relevant metals through several sources, including diet, polluted air, occupational inhalation, and cigarette smoking.

It is learned that in recent years, the occurrences of heavy metal-contaminated food (e.g., Cd rice) due to water/land pollution have increased significantly on a global scale. Thus, in order to reduce the risk of toxic metals, more systematic research on food, workplace, and environmental safety is required. Meanwhile, there are several methods that may help us reduce our exposure to heavy metals, and the most critical one is that we control and, if possible, eliminate the pollution sources. From an agricultural and industrial aspect, it is important that we rationalize the usage of fertilizers and pesticides and strictly prohibit the discharge of industrial wastes. From a government aspect, relevant departments shall formulate and improve the workplace and environmental regulations and standards, strengthen supervision, and raise public awareness of environmental protection. From a personal daily life aspect, we can use a drinking water filtration system to filter heavy metals, take precautions at work, and avoid going to or living in highly polluted areas.

**Fig. 2:**
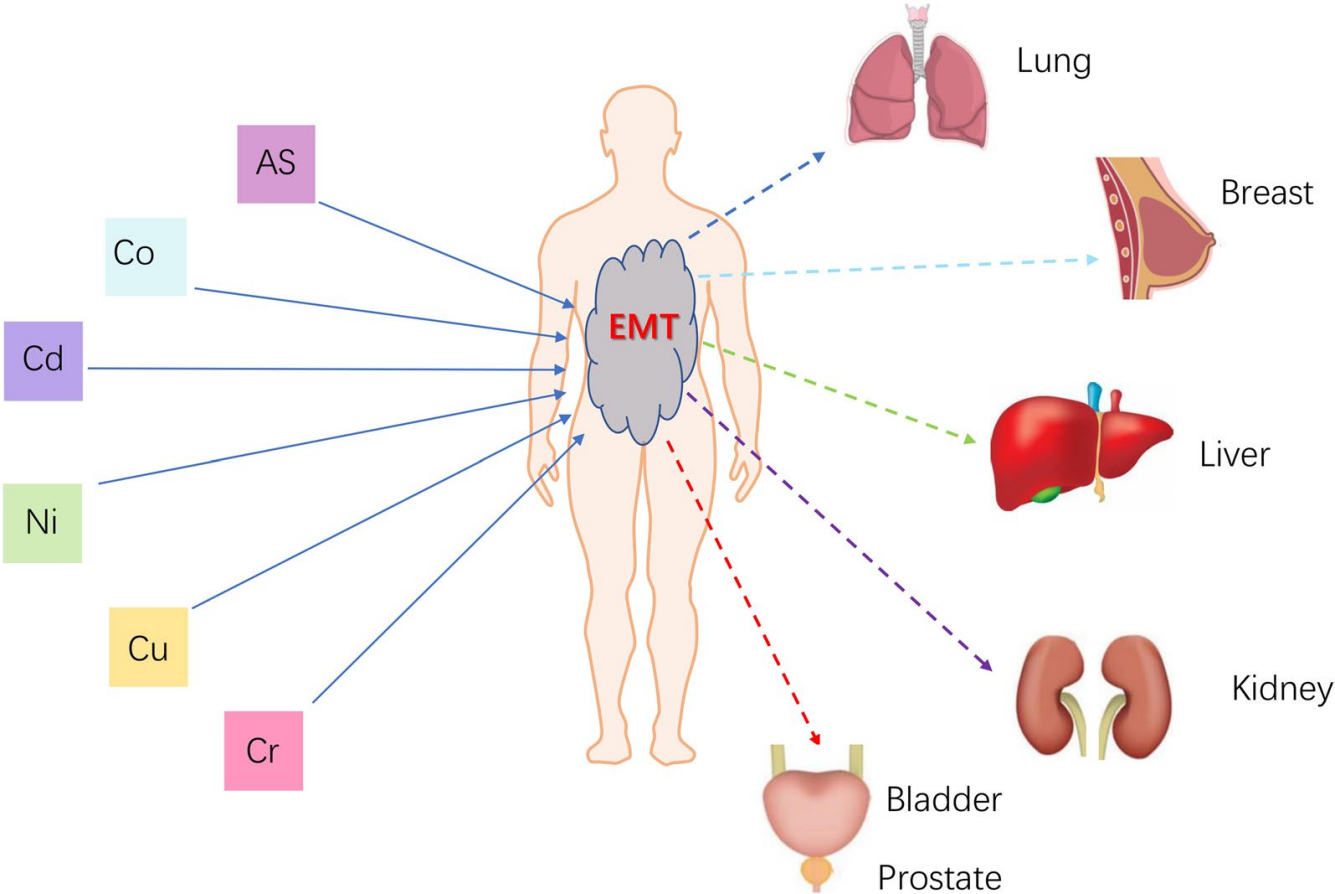
Health risks associated with the six heavy metals reviewed in this article. Chronic exposure to these metals could promote EMT and the development of cancers in the lung, breast, liver, kidney, bladder, and prostate

The cytotoxicity and carcinogenicity of heavy metals are complex, and recent studies have demonstrated that some of these metals can induce EMT in both normal and cancerous cells, leading to increased cancer risk (as shown in Fig. 2). However, as elaborated in this review, the dual role of certain heavy metals in cancer has also been observed—these heavy metals (e.g., AS, Cd, and Cu) exhibit anticancer properties, suggesting the opportunity for them to be applied in cancer therapy.

## Conclusions

In recent years, knowledge in the field of EMT has continued to expand. Even though the contribution of metals and nutrients in human cancer has been well recognized for many years, it is now becoming clear that certain metals are able to impact the process of EMT. In this review, we provide a deeper and more comprehensive picture of the effects of six toxicologically relevant metals (AS, Cd, Co, Cr, Ni, and Cu) in EMT. We also show that most of the time, heavy metals act more likely as demons by promoting EMT, while sometimes they could act more likely as angels by inhibiting EMT (summarized in Table 2). Overall, the toxicity of heavy metals is expected to be decided by the dose, route, and chemical species of exposure, together with the nutritional status, gender, age, and genetics of exposed individuals. In conclusion, this review reminds the public that we should avoid or reduce the chance of exposure to toxic heavy metals in our daily lives as exposure to these metals may promote EMT and induce malignant transformation of cells.

**Table 2:** Toxicologically relevant metals-induced EMT marker alterations

Action	Metal	Studying model/cell line	Dose characteristics	Molecules/Signaling pathway	The expression of EMT markers	Refs.
Promotes EMT	AS	• HBE cells	• 2.5 μM of NaAsO_2_ for 16 weeks	–	AS decreases the level of E-cadherin; increases the level of vimentin and ZEB1/ZEB2	[21]
		• HBE cells	• 1.0 μM NaAsO_2_ for 15 weeks	AS activates HIF-2α-dependent transcriptional activity	AS decreases the level of E-cadherin; increases the level of vimentin, ZEB1/ZEB2, and twist	[22]
		• HBE cells	• 1.0 μM NaAsO_2_	AS induces up-regulation of miR-21	AS upregulates the expression of twist	[23]
		• HaCaT cells	• 1.0 μM NaAsO_2_	AS activates NF-κB signal pathway	AS decreases the level of E-cadherin; increases the level of vimentin and SNAI1	[24]
		• HaCaT cells	• 1.0 μM NaAsO_2_	AS enhances miR-21 levels by IL-6 activation of the STAT3 signal pathway	AS decreases the level of E-cadherin; increases the level of vimentin	[25]
		• HaCaT cells	• 1.0 μM NaAsO_2_ for 0, 10, 20, 30, or 40 passages	–	AS induces down-regulation of E-cadherin and up-regulation of vimentin, ZEB1, twist, and SNAI1	[26]
		• HaCaT cells	• 1.0 μM NaAsO_2_ for 0, 10, 20, 30, or 40 passages	AS increases miR-21 and decreases PTEN levels, which then activates AKT signaling	AS decreases the level of E-cadherin; increases the level of vimentin	[27]
		• HBE cells	• 1.0 μM NaAsO_2_ for 0, 10, 20, or 30 passages	AS induces secretion of IL-6 and activates STAT3 signaling, which upregulates miR-21	AS decreases the level of E-cadherin; increases the level of N-cadherin and vimentin	[28]
		• HBE cells	• 2.5 μM of NaAsO_2_ for 16 weeks	–	AS decreases the level of E-cadherin; increases the level of vimentin	[29]
		• BEAS-2B cells	• Chronic treatment: 0.25 µM NaAsO_2_ for 16 weeks; acute treatment: 2.5 µM for 48 h	AS induces EMT likely via activation the MEK/ERK1/2 signaling	AS decreases the expression of E-cadherin; increases the expression of vimentin, ZEB1, and SNAI1	[30]
		• BEAS-2B cells	• Chronic treatment: 0.25 μM As_2_O_3_ for 10 and 20 weeks; acute treatment: 5 μM As_2_O_3_ for 0, 6, 12, and 24 h	–	AS decreases the expression of E-cadherin; increases the expression of vimentin, ZEB1, MMP-3, MMP-9, and β-catenin	[31]
		• L-02 cells	• 2.0 μM NaAsO_2_ for 0−30 passages	–	AS decreases the expression of E-cadherin; increases the expression of N-cadherin and α-SMA	[33]
		• L-02 cells	• 2.0 μM NaAsO_2_ for 0−30 passages	–	AS decreases the expression of E-cadherin; increases the expression of SNAI1and vimentin	[34]
		• HPL-1D cells	• 2 µM NaAsO_2_ for 38 weeks	AS increases the expressions of KRAS, ERK1/2, p-ERK, and AKT1	AS decreases the expression of E-cadherin; increases the expression of vimentin and MMP2	[35]
		• SV-HUC-1 cells	• 0.5 μM NaAsO_2_ for 40 weeks	AS increases the expression of HER2, which induces EMT via MAPK, AKT, and Src/STAT3 signaling pathways	AS decreases the expression of E-cadherin; increases the expression of vimentin and SNAI1	[36]
		• HK-2 cells	• 100 pg/mL and 10 ng/ mL NaAsO_2_ for 72 h for acute treatment, 2 months for chronic treatment	–	AS increases the expression of N-cadherin and vimentin	[38]
		• Caco2 and HCT116 cells	• 1 and 0.1 µM of NaAsO_2_ for short-term (36 h) and long-term (20 days) treatment	–	AS decreases the expression of E-cadherin; increases the expression of N-cadherin, FIB1, and vimentin	[39]
		• NHEK/SVTERT3‑5 cells	• 0.05, 0.1, and 0.25 µM of ATO for short-term treatment (72 h) and chronic exposure (6 months)	–	AS decreases the expression of keratin-14, ZO-1, and E-cadherin; increases the expression of TCF8/ZEB1 and SNAI2	[40]
		• HeLa cells	• 0.5 µM NaAsO_2_ for about 45 days	–	AS decreases the expression of β-catenin, claudin-1, claudin-3, and ZO-1; increases the expression of SNAI1, SNAI2, and vimentin	[41]
	Cd	• Female ApoE knockout mice	• 100 mg/L of CdCl_2_ drinking water for 12 weeks	Cd induces transcriptional activation of the Wnt pathway	Cd increases the expression of collagen I, fibronectin and twist	[61]
		• Caki-1, 786-O, and 769-P cells	• 0.1 and 0.5 μM CdCl_2_ for 24 h	Cd activates the cAMP/PKA-COX2 signaling	Cd decreases the expression of E-cadherin, increases the expressions of N-cadherin and vimentin	[59]
		• A549 and BEAS-2B cells	• 10 or 20 μM CdCl_2_ for 9−15 weeks	Cd activates Notch1 signaling, which then activates HIF-1α and IGF-1R/AKT/ERK/S6K1 signaling pathways	Cd decreases the expression of E-cadherin; increases the expression of N-cadherin and vimentin	[60]
		• BEAS-2B and BEP2D cells	• 0, 2.5, 5, and 10 μM CdCl_2_ for 72 h	Cd downregulates miR-30 family miRNAs	Cd decreases the expression of E-cadherin and increases the expressions of ZEB1 and vimentin	[55]
		• BEAS-2B cells	• 5−10 μM of CdCl_2_ for 48 h	–	Cd decreases the expression of E-cadherin, EPCAM, and KRT7; increases the expression of N-cadherin, integrin β1/β3, vimentin, and S100A11	[53]
		• MCF10A and hTERT-HPNE cells	• MCF10A: 2.5 µM CdCl_2_ for 40 weeks; hTERT-HPNE: 1 µM CdCl_2_ for 30 weeks	–	Cd decreases the expression of E-cadherin and increases the expressions of N-cadherin and vimentin	[56]
		• MCF10A, MDA-MB-231, HCC 1937 and HCC 38 cells	• 1 or 3 μM CdCl_2_ for 4 weeks	–	Cd decreases the expression of E-cadherin and claudin-1 and increases the expressions of N-cadherin and vimentin	[57]
		• Triple-negative MDA-MB-231 cells	• 1−3 μM CdCl_2_ for short-term treatment (24 h) and long-term treatment (8 weeks)	–	Cd decreases the expression of E-cadherin; increases the expression of N-cadherin, twist, and SNAI2	[58]
	Co	• MiaPaCa2 cells	• 0.08 mM CoCl_2_ for 24 h	Co induces the expression of HIF-1α, activates Notch1 signal	Co decreases the expression of E-cadherin; increases the expression of N-cadherin and SNAI1	[66]
		• MCF7 and MDA-MB-231cells	• 200 µmol/L CoCl_2_ for 24, 48, and 72 h	–	Co decreases the expression of E-cadherin; increases the expression of vimentin, MMP2, and MMP9	[70]
		• MCF7 and MDA-MB-231cells	• 300 or 450 µM CoCl_2_ for 72 h	–	Co decreases the expression of E-cadherin; increases the expression of N-cadherin and vimentin	[71]
		• TE-1 and EC-1 cells	• 100 µmol/L CoCl_2_ for 12 or 24 h	Co activates STAT3 and upregulates the expression of HIF-1α	Co decreases the expression of E-cadherin; increases the expression of N-cadherin and vimentin	[67]
		• HepG2 cells	• 200 µmol/L CoCl_2_ for 12 or 24 h	Co increased HIF-1α and COX-2 expression	Co decreases the expression of E-cadherin; increases the expression of SNAI1and vimentin	[68]
		• A549 and PC9 cells	• 100 µmol/L CoCl_2_ for 24−48 h	Co increases Netrin-1 expression and activates the PI3K/AKT pathway	Co decreases the expression of E-cadherin; increases the expression of vimentin	[69]
		• M139 and M214 cells	• 100 μM CoCl_2_ for 16 or 36 h	–	Co decreases the expression of E-cadherin; increases the expression of N-cadherin	[73]
		• LO2 cells	•100 μM CoCl_2_ for 24 or 72 h	Co activates TGF-β/Smad signaling	Co decreases the expression of E-cadherin; increases the expression of α-SMA, vimentin, N-cadherin, fibronectin, and SNAI1	[74]
		• SRA01/04 cells	• 150 μM CoCl_2_	Co induces the expression of HIF-1α and Notch1	Co decreases the expression of E-cadherin; increases the expression of SNAI1	[75]
	Cr	• 121 prostate tumor serum samples; six-week-old immunodeficient (BALB/c nude) male mice; PC3 cells	• Mice: given water containing K_2_CrO_4_ (5 μg/mL) for 14 days; cells: 0.4 µM K_2_CrO_4_ for 48 h	–	Cr (VI) decreases the expression of E-cadherin; increases the expression of N-cadherin and SNAI1	[80]
		• BEAS-2B, CrTF1, CrTF2, and A549 cells	• 0.5 μM K_2_Cr_2_O_7_ for 3–10 weeks	–	Cr (VI) decreases the expression of E-cadherin; increases the expression of vimentin	[81]
		• HK-2 cells	• 0−2 μM K_2_Cr_2_O_7_ for 1−72 h	–	Cr (VI) increases the expression of paxillin, vimentin, and α-SMA	[82]
	Ni	• Eight-week-old female immunodeficient nude mice; BEAS-2B and A549 cells	• Mice: 0, 20 or 100 mg NiCl_2_/kg/day by oral gavage for 60 days; cells: 0, 0.25, 0.5 mM and 0, 0.5, 1 mM NiCl_2_ respectively for 48 h;	Ni increases miR-4417 expression	Ni decreases the expression of E-cadherin; increases the expression of fibronectin	[86]
		• BEAS-2B cells	• Chronic treatment:100 μM NiCl_2_ for 6 weeks; acute treatment: 500 μM NiCl_2_ for 72 h	Ni suppresses the expression of ZEB1’s repressors miR-200/205	Ni decreases the expression of E-cadherin and claudin 1; increases the expression of fibronectin1 and ZEB1	[87]
	Cu	• 240 ICR mice	• Mice: 10, 20, or 40 mg CuSO_4_/kg by intragastric administration	Cu activates TGF-β1/Smad pathway and MAPKs pathways	Cu decreases the expression of E-cadherin; increases the expression of twist and vimentin	[93]
Inhibits EMT	AS	• Immortalized epicardial cells	• 1.34 μM As_4_S_4_ or 0.134 μM MMA (III) for 24 h or 48 h	AS and MMA (III) block Smad2/3, Erk1/2, and Erk5 phosphorylation	AS increases the expression of E-cadherin; decreases TGFβ2, TβRIII, SNAI1, and MMP2	[47]
		• Mca-Rh7777 cells	• 2 μM ATO for 24 or 48 h	–	ATO increases the expression of E-cadherin; decreases E-cadherin, vimentin, and twist	[44]
		• SW1353, OUMS-27, and HCS-2/8 cells	• 1.5 μM ATO for 48 h	ATO upregulates the expression of miR-125b	ATO increases the expression of E-cadherin; decreases the expression of N-cadherin, vimentin, and SNAI2	[45]
		• SMMC-7721, Huh7, MHCC97H, HCCLM3, and L02 cells	• 2 μM ATO	–	ATO increases the expression of E-cadherin; decreases the expression of N-cadherin and vimentin	[46]
		• AGS cells	• 5 or 10 μM ATO for 48 h	ATO induces SHP-1 expression and attenuates p-JAK2/ p-STAT3	ATO increases the expression of E-cadherin; decreases the expression of SNAI1	[43]
		• Immortalized murine epicardial cells	• 1.34 − 6.7 μM NaAsO_2_ for 18 h	AS blocks the canonical TGFβ signaling	AS decreases the expression of TGFβ2, TBRIII, SNAI1, and Has2	[48]
	Cd	• Adult mammary stem cells	• 0.25 and 2.5 μM CdCl_2_ for 7–10 days	–	Cd decreases the expression of ZEB1, vimentin, and TGFBI	[62]
	Cu	• 7−8-week-old male BALB/c nude mice; Hep3B and HepG2 cells	• Mice: 9.6 mg/kg Copper (II) D-gluconate by injection into the right flank twice a week for 29 days; cells: 0.1 μM Cu	Cu down-regulates NF-κB and TGF-β signaling	Cu decreases the expression of MMP2 and SNAI2; increases the expression of E-cadherin	[96]

## Data Availability

All data generated or analyzed during this study are included in this published article.

## Abbreviations

AS: Arsenic
ATO: Arsenic trioxide
BMP: Bone morphogenetic protein
Cd: Cadmium
Co: Cobalt
Cr: Chromium
Cu: Copper
DSF: Disulfiram
EMT: Epithelial–mesenchymal transition
HBE: Human bronchial epithelial
HIF-1α: Hypoxia-inducible factor-1α
MMA: Monomethylarsonous acid
TGF-β: Transforming growth factor β
uPAR: Urokinase-type plasminogen activator receptor
Ni: Nickel
